# Norepinephrine in the Medial Pre-frontal Cortex Supports Accumbens Shell Responses to a Novel Palatable Food in Food-Restricted Mice Only

**DOI:** 10.3389/fnbeh.2018.00007

**Published:** 2018-01-26

**Authors:** Emanuele Claudio Latagliata, Stefano Puglisi-Allegra, Rossella Ventura, Simona Cabib

**Affiliations:** ^1^Fondazione Santa Lucia, IRCCS, Rome, Italy; ^2^Daniel Bovet Department of Psychology and Center, Sapienza Università di Roma, Rome, Italy

**Keywords:** addiction, incentive motivation, novelty response, motivational circuits, salient stimuli, stress

## Abstract

Previous findings from this laboratory demonstrate: (1) that different classes of addictive drugs require intact norepinephrine (NE) transmission in the medial pre Frontal Cortex (mpFC) to promote conditioned place preference and to increase dopamine (DA) tone in the nucleus accumbens shell (NAc Shell); (2) that only food-restricted mice require intact NE transmission in the mpFC to develop conditioned preference for a context associated with milk chocolate; and (3) that food-restricted mice show a significantly larger increase of mpFC NE outflow then free fed mice when experiencing the palatable food for the first time. In the present study we tested the hypothesis that only the high levels of frontal cortical NE elicited by the natural reward in food restricted mice stimulate mesoaccumbens DA transmission. To this aim we investigated the ability of a first experience with milk chocolate to increase DA outflow in the accumbens Shell and c-fos expression in striatal and limbic areas of food–restricted and *ad-libitum* fed mice. Moreover, we tested the effects of a selective depletion of frontal cortical NE on both responses in either feeding group. Only in food-restricted mice milk chocolate induced an increase of DA outflow beyond baseline in the accumbens Shell and a c-fos expression larger than that promoted by a novel inedible object in the nucleus accumbens. Moreover, depletion of frontal cortical NE selectively prevented both the increase of DA outflow and the large expression of c-fos promoted by milk chocolate in the NAc Shell of food-restricted mice. These findings support the conclusion that in food-restricted mice a novel palatable food activates the motivational circuit engaged by addictive drugs and support the development of noradrenergic pharmacology of motivational disturbances.

## Introduction

Dysfunctional processing of motivationally salient stimuli has been proposed as trans-diagnostic phenotype of behavioral disturbances (Robinson and Berridge, 2001; Kapur et al., 2005; Sinha and Jastreboff, 2013; Winton-Brown et al., 2014; Nusslock and Alloy, 2017). Thus, uncovering neurobiological mechanisms of dysfunctional motivation represents a major challenge for basic research.

Although dopamine (DA) transmission in the Nucleus Accumbens Shell (NAc Shell) plays a paramount role in motivation (Di Chiara and Bassareo, 2007; Cabib and Puglisi-Allegra, 2012; Berridge and Kringelbach, 2015), severe impairment of NAc DA transmission does not always prevent development or expression of motivated responses (Nader et al., 1997). Moreover, pharmacological blockade of DA receptors in the NAc Shell disrupts expression of appetitive/avoidant responses to natural incentives promoted by local antagonism of glutamate receptors, but not those promoted by stimulation of GABAergic transmission (Faure et al., 2008; Richard et al., 2013). Finally, DA and opioids are independently involved in food motivation depending on the organism’s state (Bechara and van der Kooy, 1992; Baldo et al., 2013; Fields and Margolis, 2015). These findings support the involvement of different brain circuits in motivation and suggest the hypothesis that dysfunctional motivation could be associated to engagement of a specific brain circuit.

The engagement of NAc in motivational processes is controlled by the medial pre Frontal Cortex (mpFC; Richard and Berridge, 2013; Fiore et al., 2015; Pujara et al., 2016; Quiroz et al., 2016) and frontal cortical norepinephrine (NE) and DA transmission modulate DA release in the NAc Shell in opposite ways. Thus, increased DA transmission in the mpFC constraints mesoaccumbens DA release elicited by stress and novel palatable foods (Deutch et al., 1990; Doherty and Gratton, 1996; Pascucci et al., 2007; Bimpisidis et al., 2013), whereas enhanced NE transmission is responsible for the increase of DA in the NAc Shell promoted by different classes of addictive drugs and by acute stress challenge (Darracq et al., 1998; Ventura et al., 2003, 2005, 2007; Nicniocaill and Gratton, 2007; Pascucci et al., 2007). The observation that mpFC NE-dependent activation of mesoaccumbens DA characterizes the brain response to two known pathogens, i.e., stress and addictive drugs, suggests that engagement of this circuit could increase the risk of dysfunctional motivation. In line with this view, selective depletion mpFC NE prevents both the increase of DA outflow in the NAc and the development of conditioned place preference induced by addictive drugs (Ventura et al., 2003, 2005, 2007).

Enhanced mesoaccumbens DA release promoted by either acute stress challenge (Nicniocaill and Gratton, 2007) or amphetamine administration (Darracq et al., 1998) is selectively prevented by blockade of the low affinity alpha1 adrenergic receptors that are activated by high concentrations of frontal cortical NE (Ramos and Arnsten, 2007). These findings support the view that both addictive drugs and stress activate mesoaccumbens DA release by promoting a large increase of NE in mpFC. Recent evidence indicates that food-restricted mice respond to the first experience of a palatable food (milk chocolate) with a significantly larger increase of mpFC NE then *ad libitum* fed mice. Moreover, although both food restricted and free-fed mice develop conditioned preference for a context paired with milk chocolate, only in the formers this response requires intact frontal cortical NE transmission (Ventura et al., 2008). These findings suggest the hypothesis that in food restricted mice the experience of a novel palatable food engages the motivational circuits typically observed in animals challenged by addictive drugs. To test this hypothesis the following experiments were evaluated: (1) whether milk chocolate elicits an mpFC NE-dependent DA release in the NAc Shell of food-restricted mice; and (2) whether the first experience of milk chocolate promotes a different pattern of c-fos expression in limbic and striatal brain regions of *ad libitum* fed and food-restricted mice.

## Materials and Methods

### Animals and Housing

Male mice of the inbred C57BL/6JIco strain (Charles River, Como, Italy), 8–9 weeks old at the time of the experiments, were housed as previously described and maintained in a 12 h/12 h light/dark cycle (light on between 07.00 a.m. and 07.00 p.m.). Each experimental group consisted of 5–8 animals. All animals were treated in accordance with the principles expressed in the Declaration of Helsinki. All experiments were carried out in accordance with Italian national law (DL 116/92 and DL 26/2014) on the use of animals for research based on the European Communities Council Directives (86/609/EEC and 2010/63/UE), and approved by the ethics committee of the Italian Ministry of Health (license/approval ID #: 10/2011-B and 42/2015-PR).

Mice were individually housed and assigned to different feeding regimen, namely either receiving food *ad libitum* (FF) or subjected to food-restriction regimen (FR). FR mice received food once daily (07.00 p.m.) in a quantity adjusted to induce a loss of 15% of the original body weight. In the FF condition, food was given once daily (07.00 p.m.) in a quantity adjusted to exceed daily consumption (17 g; Ventura and Puglisi-Allegra, 2005; Ventura et al., 2008). Differential feeding regimen started 4 days before experiments.

### Drugs

Zoletil 100, Virbac, Milano, Italy (tiletamine HCl 50 mg/ml + zolazepam HCl 50 mg/ml) and Rompun 20, Bayer S.p.A Milano, Italy (xylazine 20 mg/ml), purchased commercially, were used as anesthetics, 6-hydroxydopamine (6-OHDA) and GBR 12909 (GBR), were purchased from Sigma (Sigma Aldrich, Milan, Italy). Zoletil (30 mg/kg), Rompun (12 mg/kg) and GBR (15 mg/Kg) were dissolved in saline (0.9% NaCl) and injected intraperitoneally (i.p.) in a volume of 10 ml/kg. 6-OHDA was dissolved in saline containing Na-metabisulfite (0.1 M).

### Stimuli

A piece of milk chocolate (1 g, Milka© : Fat = 29.5%; Carbs 58.5%; Proteins 6.6%) was used as palatable food in all experiments (MC). A piece of Lego© of the same size was used to control for stimulus novelty in the fos experiments and in conditioned place preference (CPP; OBJ). FF mice consumed 0.1 ± 0.05 g of MC and FR mice 0.7 ± 0.1 (*p* < 0.01, *t*-test) in the 40 min of exposure, regardless of experimental condition.

### NE Depletion in the mpFC

Animals were anesthetized with Zoletil and Rompun, then mounted in a stereotaxic frame (David Kopf Instruments, Tujunga, CA, USA) equipped with a mouse adapter. Mice were injected with GBR (15 mg/Kg, ip) 30 min before the 6-OHDA micro-injection in order to protect dopaminergic neurons. Bilateral injection of 6-OHDA (1.5 μg/0.1 ml/ 2 min for each side) was made into the mpFC (coordinates: +2.52 AP; ±0.6 L; −2.0 V with respect to bregma (Franklin and Paxinos, 2001), through a stainless steel cannula (0.15 mm outer diameter, UNIMED, Switzerland), connected to a 1 μl syringe by a polyethylene tube and driven by a CMA/100 pump (NE depleted group). The cannula was left in place for an additional 2 min after the end of the infusion. Sham animals were subjected to the same treatment, but received intracerebral vehicle. Note that in previous experiments we observed no significant difference between Sham-treated and naïve animals in basal or pharmacological/natural stimuli-induced prefrontal NE or DA outflow or in CPP or conditioned place aversion (CPA) test (Ventura et al., 2005, 2007; Pascucci et al., 2007), thus ruling out the action of GBR on observed effects in present experiments.

In all experiments animals were used 7 days after surgery.

NE and DA tissue levels in the mpFC were assessed, as previously described (Ventura et al., 2003, 2005, 2007), to evaluate the extent of depletion. In the microdialysis experiments mice were killed by decapitation to collect tissue samples from mpFC when DA levels in the NAc Shell returned to baseline (120 min after the first sampling). In the case of c-fos experiments, the frontal pole was excised immediately before the brain immersion in formalin (see “Immunostaining and Image Analyses” section). Finally, two groups (sham-depleted and NE-depleted) of unhandled mice were sacrificed 10 days after surgery to evaluate NE and DA tissue levels in both the mpFC and NAc Shell. The latter group of mice was added to rule out a subcortical spill over of the neurotoxin.

### Microdialysis

Anesthesia and surgical set are the same as described for NE depletion. Mice were implanted unilaterally with a guide cannula (stainless steel, shaft OD 0.38 mm, Metalant AB, Stockholm, Sweden) in the NAc Shell (Ventura et al., 2003, 2005, 2007). The 4.5 mm-long guide cannula was fixed with epoxy glue; dental cement was added for greater stability. The coordinates from bregma (measured according to Franklin and Paxinos, 2001) were: +1.60 anteroposterior and 0.6 lateral. The probe (dialysis membrane length 1 mm, o.d. 0.24 mm, MAB 4 cuprophane microdialysis probe, Metalant AB) was introduced 24 h before microdialysis experiments. Animals were lightly anesthetized to facilitate manual insertion of the microdialysis probe into the guide cannula and were then returned to their home cages. The outlet and inlet probe tubing was protected by locally applied parafilm. The membranes were tested for *in vitro* recovery of DA (relative recovery (%): 10.7 ± 0.82%) on the day before use in order to verify recovery.

The microdialysis probe was connected to a CMA/100 pump (Carnegie Medicine Stockholm, Sweden) through PE-20 tubing and an ultra-low torque dual channel liquid swivel (Model 375/D/ 22QM, Instech Laboratories, Inc., Plymouth Meeting, PA, USA) to allow free movement. Artificial CSF (147 mM NaCl, 1 mM MgCl, 1.2 mM CaCl_2_ and 4 mM KCl) was pumped through the dialysis probe at a constant flow rate of 2 μl/min. Experiments were carried out 22–24 h after probe placement. Each animal was placed in a circular cage furnished with microdialysis equipment (Instech Laboratories, Inc.) and with home cage bedding on the floor. Dialysis perfusion was started 1 h later, at which time the mice were left undisturbed for approximately 2 h before baseline samples were collected. The mean concentration of the three samples collected immediately before testing (less than 10% variation) was taken as basal concentration.

Immediately after collection of the three baseline samples the piece of chocolate (MC) was introduced to the cage. Dialysate was collected twice over a 40 min test to keep the experience within the time limit of a CPP training session. Only data from mice with a correctly placed cannula are reported. Placements were judged by methylene blue staining. Twenty microliters of the dialysate samples were analyzed by high performance liquid chromatography (HPLC). The remaining 20 μl were kept for possible subsequent analysis. Concentrations (pg/20 μl) were not corrected for probe recovery. The HPLC system consisted of an Alliance (Waters Corporation, Milford, MA, USA) system and a coulometric detector (ESA Model 5200A Coulochem II) provided with a conditioning cell (M 5021) and an analytical cell (M 5011). The conditioning cell was set at 400 mV, electrode 1 at 200 mV and electrode 2 at −150 mV. A Nova-Pack C18 column (3.9 × 150 mm, Waters) maintained at 30°C was used. The flow rate was 1.1 ml/min. The mobile phase was as previously described (Ventura et al., 2007, 2008). The assay detection limit was 0.1 pg.

### Immunostaining and Image Analyses

FF and FR mice, either Sham or NE-depleted, were individually exposed to an empty cage, similar to the home-cage but without food or water, 1 h daily for four consecutive days to reduce c-fos activation promoted by novel environment. On the 5th day a novel stimulus (MC or OBJ, see “Stimuli” section for details) was placed in the test cage before the mouse. Mice were left with the stimulus for 40 min, to match duration of training sessions in CPP and of dialysate collection, then were removed and left in their home cages for the following 20 min before killing by decapitation. This procedure was adopted because of previous and preliminary data indicating that in mice 60 min are required for induced accumulation of c-fos proteins (Conversi et al., 2006; Colelli et al., 2010, 2014).

Following removal of the frontal pole, to be used for evaluation of NE depletion, brains were immersed in chilled 10% neutral buffered formalin and stored overnight and then cryoprotected in 30% sucrose solution at 4°C for 48 h (Conversi et al., 2004; Paolone et al., 2007; Colelli et al., 2010, 2014). Frozen coronal sections (40 μm thickness) were cut through whole brain with a sliding microtome and then immunolabeled with immunoperoxidase method as previously described (Conversi et al., 2006; Colelli et al., 2010, 2014). Rabbit anti- c-fos (1/20,000; Oncogene Sciences) was used as primary antibody and secondary immunodetection was performed with a biotinylated antibody (1:1000 goat anti-rabbit, Vector Laboratories Inc., Burlingame, CA, USA). Peroxidase labeling was obtained by standard avidin–biotin procedure (Vectastain ABC elite kit, Vector Laboratories, diluted 1:500) and chromogenic reaction was developed by incubating sections with metal-enhanced DAB (Vector Laboratories). Immunohistochemical analyses of tissue samples obtained from FF and FR mice were performed in different batches.

Sections were analyzed using a Nikon Eclipse 80i microscope equipped with a Nikon DS-5M CCD camera as previously described (Conversi et al., 2006; Colelli et al., 2010, 2014). Specimens were subjected to quantitative image analysis using the public domain image analysis software IMAGEJ 1.38 g for Linux (Abramoff et al., 2004). Immunoreactive nuclei density was measured and expressed as number of nuclei/0.1 mm^2^.

### Place Conditioning

Behavioral experiments were performed using a place conditioning apparatus (Cabib et al., 2000; Ventura et al., 2003, 2008). The apparatus comprised two gray Plexiglas chambers (15.6 × 15.6 × 20 cm) and a central alley (15.6 × 5.6 × 20 cm). Two sliding doors (4.6 × 20 cm) connected the alley to the chambers. In each chamber two triangular parallelepipeds (5.6 × 5.6 × 20 cm) made of black Plexiglas and arranged in different patterns (always covering the surface of the chamber) were used as conditioned stimuli. The training procedure for place conditioning has been described previously (Cabib et al., 2000; Ventura et al., 2003, 2008). Briefly, on day 1 (pretest), mice were free to explore the entire apparatus for 20 min. During the following 8 days (conditioning phase) mice were confined daily for 40 min alternately in one of the two chambers. For half of the animals (from FR and FF groups) one pattern was consistently paired with MC (1 g) and the other with standard food (mouse standard diet 1 g); for the other half one pattern was consistently paired with MC (1 g) and the other with OBJ.

### Statistics

Four groups of mice were used for the microdialysis experiment: FF sham, *n* = 7; FF depleted, *n* = 5; FR sham, *n* = 6; FR depleted, *n* = 6. Data (DA output: pg/20 μl) were analyzed by two-way ANOVAs with a within factor (minute blocks following exposure to MC) and an independent factor: treatment (6-OHDA depletion or Sham depletion). The simple effect of the repeated measure (time-dependent variation of the DA levels) was also evaluated within each group.

Six groups of mice were used for the fos experiments (*n* = 5 each). Data (density of c-fos immunostained nuclei) were analyzed by two-way ANOVAs with two independent variables: novel stimulus (MC or OBJ) and treatment (6-OHDA depletion or Sham depletion). *Post hoc* analyses (Tukey’s correction) were performed whenever a significant interaction between factors was revealed.

Four groups of mice were used for the CPP experiments: 1 group of FF and 1 group of FR mice (*n* = 8 each) was trained to discriminate a compartment paired with MC and one paired with standard food chow and another group of FF (*n* = 8) and of FR (*n* = 7) mice was trained to discriminate a compartment paired with MC and one paired with an inedible object. Behavioral data (seconds spent in compartment) were analyzed by two-way ANOVAs with a within factor (compartment) and an independent factor (feeding state: FF, FR). The simple within-group effect of the compartment was evaluated within each group when a significant interaction between factors was revealed.

## Results

### Effects of 6-OHDA Infusion in the mpFC on Tissue Catecholamines Content

Tissue levels of DA and NE in Sham and NE-depleted mice from the different experiments are reported in Table 1. In all cases, local 6-OHDA infusion under GBR protection significantly reduced NE but did not affect DA levels mpFC. Levels of NE and DA in the NAc Shell were also evaluated in separate groups of mice (Unhandled) to test diffusion of the neurotoxin in this brain area. The results indicate no effects of mpFC NE depletion on either DA or NE in the NAc Shell.

**Table 1:** Tissue levels of norepinephrine (NE) and dopamine (DA) in Sham and 6OHDA-treated mice.

	Free fed	Food restricted
	**Norepinephrine (ng/g wet weight ± SE)**	
	*Microdialysis (mpFC)*	
Sham	647.5 ± 28.6 (7)	639.6 ± 37.6 (6)
6-OHDA	45.2 ± 31.1 (5)*	34.8 ± 9.0 (6)*
	*Immunohistochemistry (mpFC)*	
Sham	665.5 ± 28.0 (7)	658.4 ± 45.1 (7)
6-OHDA	57.2 ± 16.5 (7)*	47.7 ± 21.9 (7)*
	*Unhandled Groups (mpFC)*	
Sham	672.2 ± 36.6 (8)	657.2 ± 22.4 (8)
6-OHDA	68.9 ± 24.3 (8)*	56.0 ± 32.1 (8)*
	*Unhandled Groups (NAc Shell)*	
Sham	743.6 ± 42.1 (8)	750.7 ± 44.6 (8)
6-OHDA	736.0 ± 51.7 (8)	764.6 ± 34.2 (8)
	**Dopamine (ng/g wet weight ± SE)**	
	*Microdialysis (mpFC)*	
Sham	181.6 ± 15.3 (7)	202.8 ± 21.0 (6)
6-OHDA	193.6 ± 29 (5)	168.4 ± 25.8 (6)
	*Immunohistochemistry (mpFC)*	
Sham	197.1 ± 16.1 (7)	187.5 ± 18.5 (7)
6-OHDA	187.1 ± 28.1 (7)	202.1 ± 18.6 (7)
	*Unhandled Groups (mpFC)*	
Sham	191.2 ± 29.8 (8)	195.2 ± 16.3 (8)
6-OHDA	166.2 ± 19.5 (8)	159.1 ± 22.0 (8)
	*Unhandled Groups (NAc Shell)*	
Sham	12646.8 ± 438.4 (8)	12876.2 ± 418.1 (8)
6-OHDA	12344.8 ± 370.1 (8)	12511.7 ± 407.1 (8)

Note: *p < 0.0001: t-test, 2-tailed.

### Experiment 1: DA Outflow in the NAc Shell of Mice Exposed to MC for the First Time

**Figure 1:**
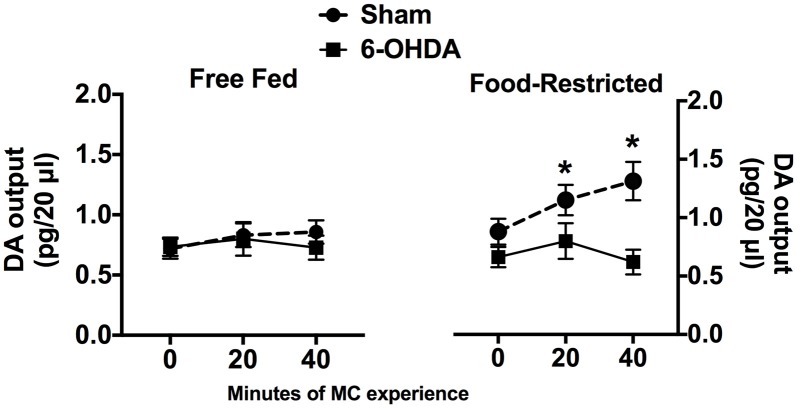
Effects of selective medial pre Frontal cortex (mpFC) norepinephrine (NE) depletion on dopamine (DA) outflow (mean pg/ 20 μl ± SEM) in the nucleus accumbens shell (NAc Shell) of Free fed (FF) and Food-restricted (FR) mice. *Significantly different (*p* < 0.05) from concentration at baseline (0).

The effects of 40 min of experience with MC on DA outflow in the NAc Shell are reported in Figure 1. Statistical analysis of data collected in FF mice did not reveal any main effect or significant interaction between factors; indeed, neither exposure to MC nor mpFC NE depletion influenced DA outflow in the NAc Shell (Figure 1, left). Instead, a significant interaction between factors was revealed for data collected in FR mice (*F*_(2,20)_ = 11.19; *p* < 0.001), due to a progressive increase of DA outflow in comparison with baseline (0) in sham-operated animals that was abolished by mpFC NE depletion (Figure 1, right).

### Experiment 2: C-fos Immunostaining in Mice Exposed to MC or to an Inedible Object for the First Time

**Figure 2:**
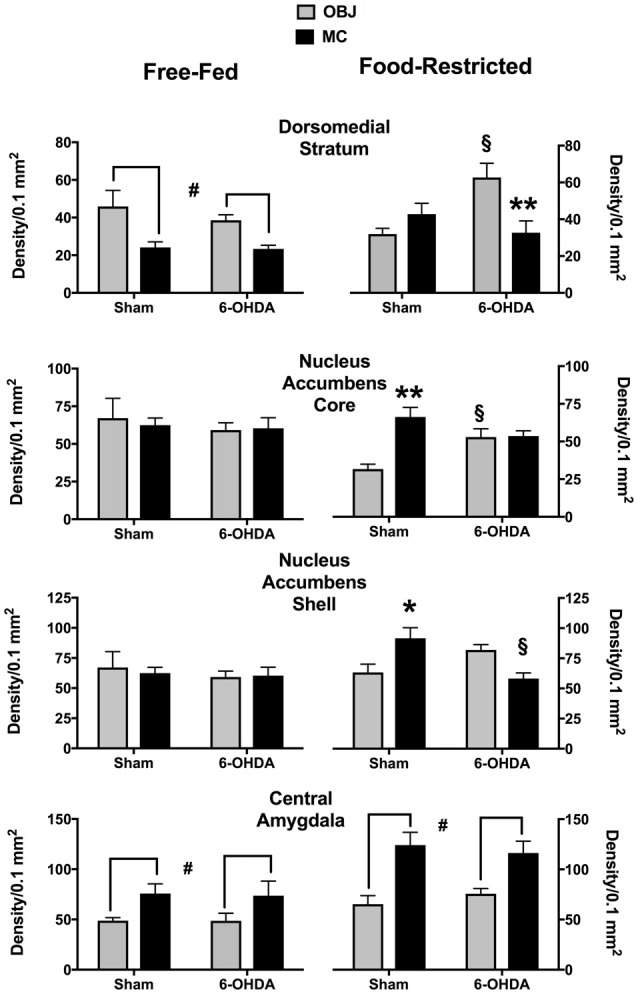
C-fos expression (mean density ± SEM) induced by the first exploration of a small piece of plastic (OBJ) or a piece of milk chocolate (MC) in different experimental conditions. ^#^Main effect of the novel stimulus (OBJ vs. MC; see text for details). *,**p < 0.05, *p* < 0.01 vs. OBJ (Tukey correction for multiple comparisons). ^§^*p* < 0.05 vs. Sham (Tukey correction for multiple comparisons).

**Figure 3:**
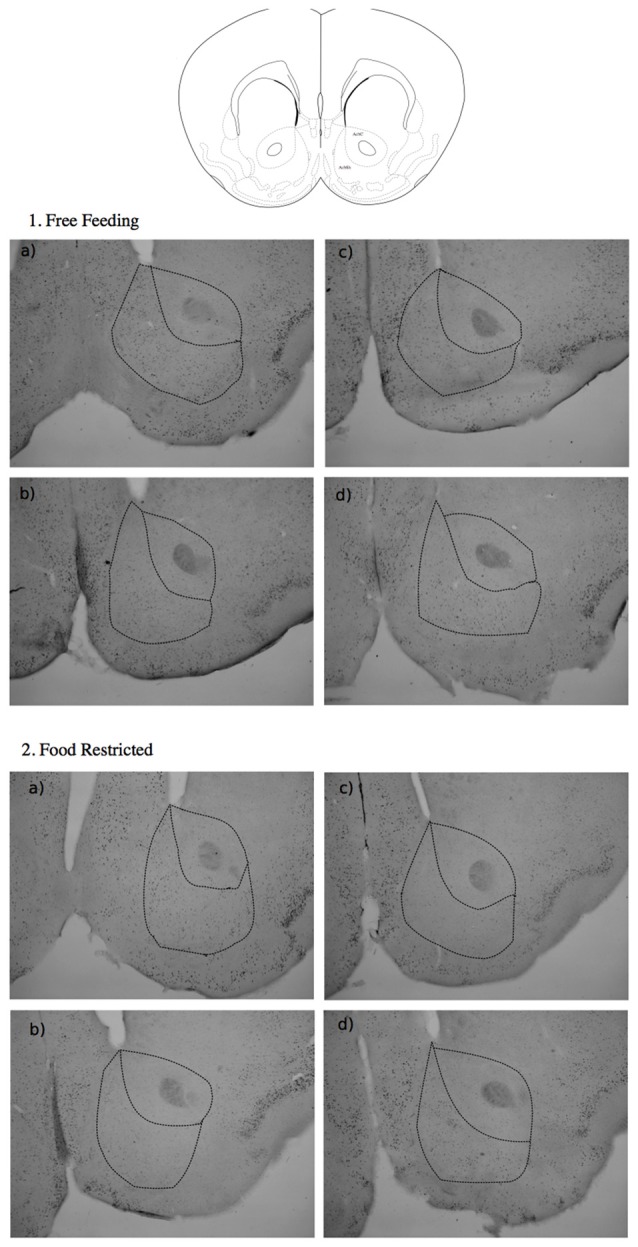
Representative images of immunostained specimens from the NAc Core and Shell of free fed (FF, top) and food-restricted (FR, bottom) mice. **(a)** Sham-depleted mice exposed to MC, **(b)** sham-depleted mice exposed to OBJ, **(c)** NE-depleted exposed to MC, **(d)** NE-depleted exposed to OBJ.

The effects of 40 min exposure to MC or to OBJ on c-fos expression are shown in Figure 2. Representative images of NAc c-fos expression in the different experimental groups are shown in Figure 3. It should be pointed out that, due to the high number of tissue samples used in these experiments, the samples collected in FF and FR mice were processed in different batches, therefore direct comparison between results obtained in these two groups is not meaningful.

Statistical analyses performed on data collected in FF mice revealed a significant main effect of the factor stimulus (MC vs. OBJ) in the Central Amygdala (CeA; *F*_(1,28)_ = 7.35; *p* < 0.05), due to higher c-fos expression in mice exposed to MC regardless of the treatment (Figure 2, bottom left), and in the Dorsomedial Striatum (DMS; *F*_(1,28)_ = 14.44; *p* < 0.001) due to higher c-fos expression in mice exposed to OBJ regardless of the treatment (Figure 2, top left). No effect of NE depletion nor significant interaction between factors stimulus and treatment were revealed by the statistical analyses of data collected in FF mice, indicating that mpFC NE depletion was totally ineffective in FF mice.

As for data collected in FR mice (Figure 2, right) statistical analyses revealed significant interactions between the factors stimulus (OBJ vs. MC) and treatment (Sham vs. NE-depleted) in the DMS (*F*_(1,24)_ = 11.5; *p* < 0.005), NAc Core (*F*_(1,24)_ = 12.28; *p* < 0.005), and NAc Shell (*F*_(1,24)_ = 16.28; *p* < 0.001). In sham-operated mice MC promoted a larger increase of c-fos immunostained nuclei then OBJ in NAc Core and Shell (Figure 2, right). This effect was not observed in NE-depleted animal due to a decrease of MC-induced c-fos expression in the NAc Shell and an increase of OBJ-induced c-fos expression in the NAc Core. In the DMS of sham-operated FR mice OBJ was unable to promote c-fos expression higher than that promoted by MC (Figure 2, top right). Frontal cortical NE depletion significantly increased c-fos expression promoted by OBJ in the DMS, thus recovery the pattern of c-fos activation observed in FF mice.

In the CeA of FR mice statistical analyses only revealed a main effect of the factor stimulus (MC vs. OBJ; *F*_(1,24)_ = 24.93; *p* < 0.0001) due to higher c-fos expression in mice exposed to MC regardless of the treatment (Figure 2, bottom right).

### Experiment 3: Conditioned Preference for MC-paired Context

**Figure 4:**
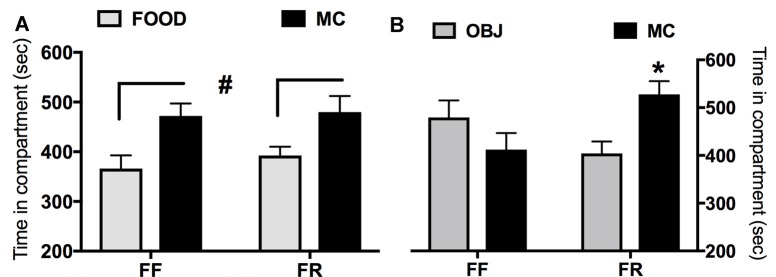
Effects of restricted feeding (FR) on conditioned preference (seconds spent in compartment ± SEM) for a context paired with milk chocolate (MC) in different experimental conditions. **(A)** Preference for the MC-paired compartment vs. the compartment paired with habitual lab chow (FOOD); **(B)** preference for MC-paired compartment vs. the compartment paired with novel inedible objects (OBJ). ^#^Main effect of the compartment (*p* < 0.05; see text for details). *Significantly different (*p* < 0.05) from time spent in the alternative compartment.

In Figure 4 are reported data from the CPP experiments. Either FR or FF mice showed significant preference for the compartment paired with MC when the other was paired with the habitual chow food (main effect of the pairing regardless of the feeding condition *F*_(1,13)_ = 12.36; *p* < 0.005; Figure 4A). Instead, when the other compartment was paired with OBJ (Figure 4B), only FR mice showed a significant preference for the MC-paired one (significant interaction between pairing and feeding condition: *F*_(1,13)_ = 5.382; *p* < 0.05).

## Discussion

Major findings of the present study are: (1) only sham-treated FR mice showed increased DA outflow in the NAc Shell during the first experience with MC; (2) only sham-treated FR mice showed MC-induced c-fos expression in the NAc Shell larger than that elicited by a novel inedible object; (3) in the DMS of FF mice and in mpFC NE-depleted FR mice a novel inedible object promoted c-fos expression larger than that promoted by the palatable food; and (4) although both FF and FR mice developed conditioned preference for MC-paired context when the other was associated with habitual food, only FR mice developed preference for the compartment paired with the palatable food when the other was associated with object novelty.

### Food Restricted but Not *ad libitum* Fed Mice Show Enhanced DA Outflow in the NAc Shell When Experiencing Milk Chocolate for the First Time and This Response Is Prevented by Depletion of Frontal Cortical NE

A first set of experiments demonstrated that initial experience with MC promotes an increase of DA outflow in the NAc Shell of FR but not FF mice. It is worth pointing out the discrepancy between present and previous results obtained in rats (Bassareo and Di Chiara, 1999), that can be easily explained by species difference as well as by differences in the type of milk chocolate used (white chocolate in the previous study: see Ventura et al., 2008 for details).

Our data also demonstrate that mesoaccumbens DA response to the novel palatable food by FR mice requires intact frontal cortical noradrenergic transmission because it was abolished by a selective depletion of frontal cortical NE. The noradrenergic depletion did not influence DA outflow in the NAc of FF mice although it has been shown to prevent the moderate increase of mpFC NE outflow elicited by MC in these mice (Ventura et al., 2008). This finding offers strong support to the view that DA outflow in NAc Shell is only controlled by large NE concentrations in mpFC.

There was no effect of mpFC NE depletion on the amount of consumed chocolate although FR mice ate significantly more MC than FF mice (see “Materials and Methods” section), these data are in line with those obtained in mice exposed to the palatable food for a much longer time (Ventura et al., 2008) and with the general observation that feeding behavior does not require enhanced mesoaccumbens DA transmission (Nicola, 2016; Boekhoudt et al., 2017).

### A First Experience of MC Promotes a Different Pattern of c-fos Expression in the Striatum of *ad libitum* Fed and Food-Restricted Mice and Frontal Cortical NE Depletion Only Influences c-fos Expression Elicited by Incentive Stimuli in Food Restricted Mice

A second set of experiments evaluated whether a first experience with MC engages different brain circuits depending on the feeding state of the organism. To this aim we evaluated the pattern of brain c-fos activation elicited by the palatable food because increasing evidence supports the use of this brain mapping strategy in rodents (Knapska et al., 2007; Ago et al., 2015; Jiménez-Sánchez et al., 2016). To control for the effect of stimulus novelty, known to activate c-fos expression in the brain (Jenkins et al., 2004; Struthers et al., 2005; Knapska et al., 2007; Rinaldi et al., 2010), we used exposure to a novel inedible object (OBJ).

The results obtained offer strong support to the tested hypothesis. Thus, only in FR mice NAc c-fos expression promoted by MC was larger than that promoted by OBJ; moreover in these mice, but not in *ad-libitum* fed mice, mpFC NE depletion selectively reduced c-fos expression elicited by MC in the NAc Shell, indicating the requirement of intact mpFC NE transmission. These findings parallel the results obtained with microdialysis and support a causal relationship between the two because of strong evidence for a major role of stimulation of DA receptors in striatal c-fos expression (Badiani et al., 1998; Barrot et al., 2000; Carr et al., 2003; Bertran-Gonzalez et al., 2008; Colelli et al., 2010; Ago et al., 2015). By contrast, a larger increase of c-fos expression in OBJ- vs. MC-exposed mice was observed in the DMS of Sham-depleted mice. A strong activation elicited by the novel inedible object in the DMS is coherent with previous findings in mice and rats (Struthers et al., 2005; Rinaldi et al., 2010) and with the main role of DMS functioning for exploration of novel objects (Durieux et al., 2012). Restricted feeding reduced OBJ-induced c-fos expression in DMS and mpFC NE depletion abolished the effect of food restriction, suggesting an inhibitory control of frontal cortical NE on the induction of c-fos expression in the DMS of FR mice. Moreover, although the first MC experience elicited a larger c-fos expression than OBJ in the NAc Core of FR mice, mpFC-NE depletion eliminated this difference by increasing c-fos expression in OBJ-exposed mice rather than by reducing c-fos expression in MC-exposed mice. Together, these findings support the hypothesis that in FR mice increased frontal cortical NE transmission enhances c-fos expression promoted by exploration of MC in the NAc Shell and inhibits c-fos expression induced by exploration of a novel inedible object in both the DMS and the NAc Core.

On the other hand, both FF and FR mice showed a larger increase of c-fos expression in the CeA when exposed to MC than when exposed to OBJ, and in both groups the response was still evident following mpFC NE depletion. The latter finding is in line with the view that induction of c-fos expression in the CeA by novel palatable tastes is mediated by gustatory afferent information from the parabrachial nuclei of the pons (Koh et al., 2003; Knapska et al., 2007). Although CeA activation by novel tastes has been proposed to mediate food neophobia: an aversive response, this interpretation has been challenged by results of lesion studies (Reilly and Bornovalova, 2005) and by the observation that stimulation of CeA μ-opioid receptors enhances incentive salience of different stimuli including palatable food (Mahler and Berridge, 2012). Moreover, there is consistent evidence for a role of the CeA in the Pavlovian appetitive conditioning and, in particular, in place conditioning (Knapska et al., 2007; Rezayof et al., 2007). Therefore, activation of CeA could contribute to mpFC NE-independent MC-induced CPP in FF mice (Ventura et al., 2008).

### Only FR Mice Develop Conditioned Preference for a Context Paired with a Novel Palatable Food When the Other Is Associated with an Inedible Novel Object

In FF mice there was no difference in NAc c-fos expression elicited by MC or OBJ. The most conservative interpretation of this finding is that the two stimuli were equally salient possibly because of their novelty. Indeed, novel objects are strong incentive for rodents (Reichel and Bevins, 2008). This interpretation could also explain why both FF and FR mice develop conditioned preference for an MC-paired context when the other is associated with habitual lab chow although only in FR mice this conditioning is prevented by mpFC NE depletion (Ventura et al., 2008). In other words, motivational salience of MC could depend on novelty in FF but not in FR mice. To test this hypothesis, we trained FF and FR mice in an apparatus that contrasted a compartment associated with the novel palatable food with one associated with novel objects. We reasoned that if novelty motivates conditioned preference for the MC paired context in FF mice no preference should be observable when a different novel stimulus is associated with the other compartment.

The results obtained strongly supported this hypothesis. Indeed, FF mice did not develop conditioned preference for the compartment associated with MC when the other was associated with object novelty although, as previously reported (Ventura et al., 2008), they showed conditioned preference for the MC-paired compartment when the other was associated with a well-known taste. By contrast, FR mice preferred the MC-associated compartment in both experimental settings supporting the conclusion that incentive salience of MC and MC-associated stimuli for these mice is unrelated to novelty. This conclusion supports the role of CeA in CPP induced by MC in FF but not in FR mice. Therefore, behavioral and c-fos findings of the present experiments converge to indicate that different brain circuits process motivational salience of the novel palatable food in the two feeding conditions.

Finally, the observation that OBJ competes with MC for place conditioning in FF but not in FR mice indicates that the motivational salience of the novel palatable food is higher in the latter group. Indeed, a previous study reported that novel objects compete with low but not with high doses of cocaine for place conditioning (Reichel and Bevins, 2010). Moreover, because the first experience of MC prompts an increase on frontal cortical NE larger in FR then in FF mice (Ventura et al., 2008) these findings support the hypothesis that the extent of frontal cortical NE release elicited by an incentive stimulus is dependent on strength of its motivational salience (Puglisi-Allegra and Ventura, 2012).

## General Conclusion and Implications

The findings of the present study support the general conclusion that a specific brain circuit involving the NAc Shell through high NE levels in mpFC is engaged by addictive drugs, stress, and by palatable food in food restricted mice. Thus, as discussed, only blockade of alpha1 receptors, sensitive to high but not moderate frontal cortical NE concentrations (Ramos and Arnsten, 2007), prevents stress- (Nicniocaill and Gratton, 2007) and amphetamine-induced mesoaccumbens DA release (Darracq et al., 1998). Seemingly, only in FR mice, characterized by a significantly larger mpFC NE response MC than FF mice (Ventura et al., 2008), the palatable food enhances DA release and c-fos expression in the NAc Shell, and this effect is prevented by selective mpFC NE depletion.

The finding that in FR mice a novel palatable food engages a brain circuit engaged by addictive drugs and stress is not surprising. Indeed, food-restricted mice and rats show addiction-like behavioral and neural phenotypes in the laboratory (Cabib et al., 2000; Carr, 2011; Campus et al., 2017) and human data indicate that restrained eating is associated to loss of control, bingeing and counterproductive weight gain, whereas severe dieting is a risk factor for binge pathology and substance abuse (Carr, 2011). Therefore, the findings of the present study support the hypothesis that high frontal cortical concentration of NE can be responsible for dysfunctional motivation through engagement of a specific brain circuit.

Dysfunctional processing of motivationally salient stimuli has been proposed as trans-diagnostic phenotype of very different disturbances (Robinson and Berridge, 2001; Sinha and Jastreboff, 2013; Winton-Brown et al., 2014; Nusslock and Alloy, 2017), including schizophrenia (Kapur et al., 2005; Velligan et al., 2006; Reckless et al., 2015). The involvement of NE transmission in psychopathology has been long known and has supported development of pharmacological treatments aimed at adrenergic receptors (Ramos and Arnsten, 2007; Borodovitsyna et al., 2017; Maletic et al., 2017). The main target of these interventions is cognitive functioning (Arnsten, 2015), although there is also evidence that NE manipulation can affect the positive symptoms associated with schizophrenia (Borodovitsyna et al., 2017; Maletic et al., 2017). To these targets, present findings add dysfunctional motivation by supporting the involvement of high frontal cortical NE transmission in this trans-diagnostic phenotype (Robinson and Berridge, 2001; Kapur et al., 2005; Sinha and Jastreboff, 2013; Winton-Brown et al., 2014; Nusslock and Alloy, 2017).

## Author Contributions

SC, ECL and SP-A planned the experiments and processed data; SC, ECL, SP-A and RV worked on the manuscript; ECL and RV performed experiments; SC wrote the manuscript.

## Conflict of Interest Statement

The authors declare that the research was conducted in the absence of any commercial or financial relationships that could be construed as a potential conflict of interest. The reviewer LP and handling Editor declared their shared affiliation.
